# The effect of psychological intervention combined with standard pharmacological therapy in patients with chronic prostatitis/chronic pelvic pain syndrome (CP/CPPS): A randomized controlled trial

**DOI:** 10.1097/MD.0000000000044110

**Published:** 2025-08-29

**Authors:** Xiu Lin, Xian-Bin Pan, Bao-Zhu Guo, Shih-Pin Lee

**Affiliations:** a Department of Public Health, International College, Krirk University, Bangkok, Thailand; b Department of Urology, Affiliated Hospital of Guilin University of Technology, Guilin, China; c Department of Surgery, Affiliated Hospital of Guangxi Normal University, Guilin, China.

**Keywords:** anxiety, depression, prostatitis course, prostatitis pharmacotherapy, psychological intervention, type III prostatitis

## Abstract

**Background:**

Chronic prostatitis/chronic pelvic pain syndrome (CP/CPPS) often coexists with anxiety and depression. This study assessed whether adding psychological intervention (PI) to standard pharmacotherapy improves patient outcomes.

**Methods:**

One hundred sixty eight adult patients diagnosed with chronic prostatitis/chronic pelvic pain syndrome (CP/CPPS) were recruited for this study. They were randomly divided into control and PI groups. All patients in both groups received a 30-day standard prostatitis pharmacotherapy regimen with levofloxacin and tamsulosin. Additionally, the 84 patients in the PI group received PIs, unlike those in the control group. The self-rating depression scale and self-rating anxiety scale were employed to assess the psychological symptoms before and 3 months after the therapeutic treatments.

**Results:**

Following treatment, the PI group exhibited significantly better recovery from symptoms of anxiety, depression, and prostatitis compared to the control group. Multiple linear regression analysis revealed that patients with longer disease durations had higher self-rating anxiety scale, self-rating depression scale, and National Institutes of Health-Chronic Prostatitis Symptom Index Scale scores. The study demonstrated that PI has positive early effects on patients with CP/CPPS. However, its efficacy decreases as the duration of the disease progresses.

**Conclusion:**

The combination of PI and conventional antibiotics is effective for CP/CPPS patients with longer disease course and more severe symptoms. Early PI is recommended to achieve better therapeutic results.

## 1. Introduction

Chronic prostatitis/chronic pelvic pain syndrome (CP/CPPS) is the most common prostate disease in men, characterized by high morbidity and recurrence rates.^[1]^ The prevalence ranges from 4.5% to 9%, with the recurrence rate reaching up to 50% in older age groups.^[2]^Prostatitis is a major global health issue, with incidences varying between 6.0% and 32.9%, and 2 peak incidence rates were observed in the 30 to 40-year and 61 to 70-year age groups.^[3]^ This condition is primarily associated with pain in the urogenital region and disturbances in urinary and sexual function.^[4,5]^ The National Institute of Health (NIH) recognizes 4 types of prostatitis: acute bacterial prostatitis (type I), chronic bacterial prostatitis (type II), CP/CPPS; type III, and asymptomatic inflammatory prostatitis (type IV).^[6]^ Type III prostatitis is notably one of the most complex urinary system diseases.^[7]^ Common genitourinary complaints in CP/CPPS include ejaculatory pain (perineal, lower abdominal, testicular, and penile), voiding disorders, and sexual dysfunction.^[8]^ Beyond physical discomfort, over 80% of CP/CPPS patients also experience serious psychological issues, such as depression, anxiety, and stress, adversely impacting their health-related quality of life.^[8–12]^ Research has found that approximately 30% to 50% of CP/CPPS patients exhibit clinically significant depressive symptoms.^[13]^ However, antibiotics are ineffective in treating CP/CPPS, with only 50% to 65% of male patients experiencing nonsignificant long-term improvement.^[10,11]^ Thus, during therapy, factors like stress frequency, stress perception, and coping strategies should be considered for both exacerbating and ameliorating CP/CPPS symptoms. The exact cause and mechanism of CP/CPPS are not fully understood. Current research mainly focuses on factors such as autoimmunity, pathogen infection, urinary dysfunction, psychological factors, and neuroendocrine elements.^[14–16]^ Due to the high comorbidity of depression in this population,^[17,18]^ stress has been suggested as a potent factor in the development, prolongation, and perpetuation of CP/CPPS symptoms.^[19]^ Mental and psychological changes can cause autonomic nerve dysfunction, leading to neuromuscular dysfunction in the posterior urethra, which exacerbates pelvic pain and urinary dysfunction.^[8]^ The severity of stress and anxiety depends on individual perceptions or subjective interpretations of causative factors, and individual coping strategies and cognitive assessments of stress affect stress progression.^[20]^

Currently, psychological intervention (PI) as a treatment for CP/CPPS is a new direction.^[21]^ Conventional medical treatment typically involves quinolones and tamsulosin for more than 6 weeks.^[22]^ Integrating mental and physical healthcare is now a key priority for clinical commissioning groups.^[23]^ This article focuses on exploring the necessity and clinical efficacy of combining PI with standard drug treatment.

## 2. Methods

### 2.1. Patients

Building on a related study by Tripp et al,^[24]^ which investigated 44 men diagnosed with CP/CPPS and their spouses, this study expanded the sample size to ensure statistical power. A total of 220 patients with CP/CPPS were retrospectively evaluated at the Affiliated Hospital of Guilin University of Technology (Guilin, China) between March 2014 and November 2020. Out of these, 52 patients were excluded from the study. The reasons for exclusion included previous diagnoses of anxiety, depression, or other psychological issues by clinical psychologists, or pelvic pain attributed to conditions such as infected prostatitis, gonorrheal urethritis, nongonococcal urethritis, chronic pelvic pain syndrome, benign prostatic hyperplasia, prostate cancer, or urinary calculi. Consequently, the study ultimately included 168 patients diagnosed with CP/CPPS based on the National Institutes of Health criteria. These patients were aged between 18 and 54 years and had experienced prostatitis symptoms for more than 3 months without improvement despite treatment with antibiotics or α-androgen receptor antagonists. The study was approved by the Ethical Review Committee of Affiliated Hospital Guilin University of Technology (Approval No. GLUTH-2014002), and informed consent was obtained and signed by each patient participating in the study.

### 2.2. Study design

A total of 168 patients with CP/CPPS were randomly allocated to the control group (CON) and PI group using a random number table method, with age-stratified randomization to ensure matched baseline characteristics between groups (*P* > .05). In the control group, 84 patients received standard prostatitis pharmacotherapy, which included twice-per-day intravenous administration of 0.2 g levofloxacin lactate (Kelun Pharmaceutical Co., Ltd., Sichuan, China) in 100 mL normal saline (Kelun Pharmaceutical Co., Ltd., Guizhou, China) for 10 days, and once-per-day oral administration of controlled-release tamsulosin hydrochloride capsules (0.2 mg/capsule, Hengrui Medicine Co., Ltd., Jiangsu, China) for 30 days. This group did not receive any PIs.

In contrast, the PI group, consisting of the other 84 patients, received the same prostatitis pharmacotherapy as the control group, but they were additionally treated with PIs. All patients in both groups completed the Chinese versions of the National Institutes of Health-Chronic Prostatitis Symptom Index Scale (NIH-CPSI), the self-rating anxiety scale (SAS), and the self-rating depression scale (SDS) before and 3 months after the therapies.^[25]^ Prior to administration, clinical psychologists provided standardized instructions to ensure patients’ comprehension of the SAS/SDS 4-point Likert scale (1 = rarely; 4 = always). The SAS, SDS, and PIs were administered by clinical psychologists at the hospital to assess the anxiety and depression levels of the CP/CPPS patients.^[26,27]^ The NIH-CPSI scores were evaluated by the hospital’s urologists to estimate the severity of prostatitis in CP/CPPS patients.

### 2.3. Psychological intervention

The PI program at the Division of Urology in the Hospital of Guilin University of Technology was initiated in 2014. These interventions, conducted by clinical psychologists at the hospital, were specifically designed for patients in the PI group suffering from CP/CPPS. Clinical psychologists were available from 12:00 am to 4:00 pm, Monday through Friday. The primary goal of the PIs was to provide emotional support and coping strategies to CP/CPPS patients. These strategies were aimed at addressing issues related to pain, discomfort, reduced quality of life, and sexual frustration, as well as enhancing patient compliance with medical treatments.

Following standard prostatitis pharmacotherapy, PIs were administered for 30 minutes every 3 days over 30 days, totaling 10 sessions for each patient in the intervention group. The PIs encompassed a range of techniques, including cognitive behavioural therapy (CBT), acceptance and commitment therapy (ACT), positive mindfulness-based interventions, a stepped care approach, educational interventions, counseling, stress management, psychosocial support, and coping strategies. These interventions aimed to alleviate psychological stress caused by symptoms such as anxiety, depression, fear, despair, hopelessness, and sexual dysfunction. Additionally, they sought to mitigate the discomfort associated with CP/CPPS and medical procedures.

Cognitive interventions in this context focus on altering negative or unrealistic thoughts, replacing them with more positive beliefs and attitudes. For example, encouraging positive self-statements like “I can get through this instead of “this is going to hurt.”^[28]^ CBT for pain management aims to lessen psychological distress and enhance physical and role functioning. It helps individuals reduce unhelpful behaviors, increase helpful behaviors, modify unhelpful thought patterns, and boost self-efficacy in managing pain.^[29]^ Behavioral interventions target negative or maladaptive behaviors, replacing them with positive and adaptive ones. An example is choosing to watch a funny movie instead of dwelling on the discomfort of a medical procedure.^[30]^ The overarching goal of PIs for CP/CPPS is to aid individuals in developing and utilizing coping mechanisms for pain management. These interventions focus on creating effective coping strategies that reduce symptoms, improve quality of life, and enhance overall well-being. Techniques may include distraction, relaxation training, deep breathing, hypnosis, rehearsing procedures in advance, positive reinforcement, making coping statements, and coaching in adaptive strategies.^[30]^

ACT, a mindfulness-based therapy, challenges conventional treatments by acknowledging that normal human mental processes can be disruptive and lead to suffering. It emphasizes understanding the patient’s desired direction in dealing with the illness and identifying obstacles to progress. ACT focuses on accepting challenging thoughts and emotions rather than attempting to control or change them, fostering a sense of security through positive thinking exercises.^[28]^ Positive thinking interventions transform the relationship individuals have with their thoughts, emotions, and feelings, encouraging CP/CPPS patients to accept and experience their present moments without judgment. Regular practice of positive thinking meditation enhances awareness and control over responses to different situations, reducing automatic responses to pain or distress and leading to more beneficial conscious choices. This approach is grounded in Jon Kabat-Zinn’s positive thinking for mindfulness-based stress reduction method.^[31]^ Formal psychometric measures are routinely used in urology and male health departments to assess the risk of anxiety and depression in CP/CPPS patients. While these measures support clinical judgment, they should be interpreted in the context of the individual’s overall assessment and treatment performance. They are useful for stratifying psychotherapy for patients. Psychological and multidisciplinary approaches to pain management are well-established and scientifically rational. Customized psychological counseling and peer communication education are provided to CP/CPPS patients alongside conventional medication. These services aim to enhance treatment adherence, boost confidence in recovery, improve understanding of CP/CPPS, and refine patients’ ability to follow the treatment regimen.

It poses a number of challenges to conventional treatments, as it is based on the fact that normal human mental processes are often somewhat disruptive and cause corresponding suffering. The main questions in this form of therapy are, “Which direction does the patient want the illness to take?” and “What is stopping the patient from making progress with the illness?” Throughout the therapy, we consistently revisit the patient’s present emotions and the exact behavioral changes they are striving to make feasible, while fostering dedication and ensuring the patient’s safety. The ACT model aims to shift focus from coping mechanisms that emphasized the ability of CP/CPPS patients to control or change their psychological experience, to embracing challenging thoughts and emotions. Positive thinking exercises during the session help to build a sense of security in CP/CPPS patients. The main distinction between this approach and therapies such as CBT is that it advocates for the patient to dissolve their thoughts themselves, instead of addressing them through evaluation.

Positive thinking interventions aim to transform the relationship that individuals have with their thoughts, emotions and feelings by encouraging CP/CPPS patients to embrace and perceive their present moment experiences without judgment or control. Therefore, practicing positive thinking and mindfulness can help individuals become more aware and in control of their responses to various situations. This ability can be honed by regularly practicing positive thinking meditation. It is beneficial to be actively aware of our habitual responses, as they are often automatic and go unnoticed. As patients with CP/CPPS develop greater awareness, their automatically-driven responses to pain or distress are reduced, which may lead to making conscious choices about how to respond in a beneficial manner. Positive thinking has been extensively employed in pain management since Jon Kabat-Zinn first introduced his method of positive thinking for mindfulness-based stress reduction.^[31]^ Formal psychometric measures routinely administered in urology and male departments to determine the level of risk for anxiety and depression in patients with CP/CPPS. Although these measures may supplement the clinical judgment of therapists involved in the care of the patient and need to be interpreted in the context of the individual’s performance in assessment and/or treatment, they can be a useful method of stratifying psychotherapy for patients. Psychological and multidisciplinary approaches to the management of pain are widely accepted and based on an established scientific rationale. Customized psychological services and peer communication education are available to patients with CP/CPPS to bolster their adherence to treatment, instill confidence in their recovery, enhance their understanding of CP/CPPS and refine their ability to execute the treatment regimen, in addition to conventional medication.

### 2.4. Statistical analysis

For graphing and statistical analysis, we employed GraphPad Prism version 10.1.0 (GraphPad Software Inc., Boston). All data were presented as mean ± standard deviation. We calculated correlation coefficients between features using Pearson correlation analysis. Differences between the 2 groups were evaluated using 2-tailed nonparametric analysis and Mann–Whitney *U* tests. We compared parameters using repeated-measures 2-way analysis of variance (ANOVA), followed by Sidak post hoc tests. These post hoc tests were conducted only if the *F*-value in the ANOVA achieved statistical significance (*P* < .05) and there was no significant variance inhomogeneity. Multiple linear regression analysis was used to model the linear relationship between predicted therapeutic effects and response parameters. A *P*-value of <.05 was considered statistically significant in all analyses.

## 3. Results

### 3.1. Demographic characteristics and clinical features of the subjects

Among the 168 patients, the mean age was 25.47 ± 9.94 years, and the average duration of prostatitis was 9.01 ± 4.63 months. Before therapy, the mean SAS of the patients was 68.13 ± 8.31; the mean SDS was 65.11 ± 9.69; and the mean NIH-CPSI score was 22.72 ± 8.73.

**Figure 1.**
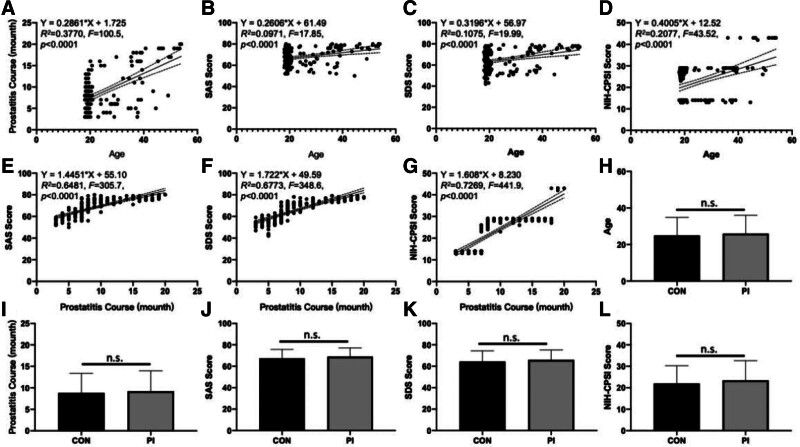
Demographic characteristics and clinical features of CP/CPPS patients in this study, comparing the CON and psychologically intervened (PI) groups. The distributions are shown for (A) the duration of prostatitis, pre-therapeutic (B) self-rating anxiety scale (SAS), (C) self-rating depression scale (SDS), and (D) National Institutes of Health chronic prostatitis Symptom Index (NIH-CPSI) scores about the age of patients (n = 168). The distributions of pre-therapeutic (E) SAS, (F) SDS, and (G) NIH-CPSI scores are presented about the duration of CP/CPPS in patients. The mean ± standard deviation for (H) age (I) duration of prostatitis, pre-therapeutic (J) SAS, (K) SDS, and (L) NIH-CPSI scores are compared between the control and PI groups (each n = 84). Solid lines represent trend lines, and dashed lines represent the 95% confidence bands of the best-fit lines. “n.s.” indicates a nonsignificant difference (*P* > .05) between the 2 groups as determined by the Mann–Whitney *U* tests. CON = control, CP/CPPS = chronic prostatitis/chronic pelvic pain syndrome, PI = psychological intervention, SAS = self-rating anxiety scale, SDS = self-rating depression scale, NIH-CPSI = National Institutes of Health-Chronic Prostatitis Symptom Index, n.s. = nonsignificant.

In the correlation analysis between age and clinical features, older patients exhibited longer prostatitis courses (*r* = 0.6140, *R*² = 0.3770, *F* = 100.5, *P* < .0001, Fig. 1A), higher pre-therapeutic SAS (*r* = 0.3116, *R*² = 0.0971, *F* = 17.9, *P* < .0001, Fig. 1B) and SDS scores (*r* = 0.3278, *R*² = 0.1075, *F* = 19.99, *P* < .0001, Fig. 1C), and more severe symptoms leading to higher pre-therapeutic NIH-CPSI scores (*r* = 0.4557, *R*² = 0.2077, *F* = 43.52, *P* < .0001, Fig. 1D). Additionally, patients with longer prostatitis durations showed higher pre-therapeutic SAS (*r* = 0.8050, *R*² = 0.6481, *F* = 305.7, *P* < .0001, Fig. 1E), SDS (*r* = 0.8230, *R*² = 0.6773, *F* = 348.6, *P* < .0001, Fig. 1F), and NIH-CPSI scores (*r* = 0.8526, *R*² = 0.7269, *F* = 441.9, *P* < .0001, Fig. 1G).

### 3.2. Difference between control and psychological groups

In the control group (n = 84), the mean age was 24.95 ± 9.94 years, the mean prostatitis duration was 8.82 ± 4.53 months, the mean pre-therapeutic SAS was 67.29 ± 8.43, the mean SDS was 64.37 ± 10.02, and the mean NIH-CPSI score was 21.99 ± 8.27. In the PI group (n = 84), the mean age was 25.99 ± 9.98 years, the mean prostatitis duration was 9.20 ± 4.75 months, the mean pre-therapeutic SAS was 68.96 ± 8.16, the mean SDS was 65.86 ± 9.35, and the mean NIH-CPSI score was 23.45 ± 9.17.

According to the Mann–Whitney *U* test, there were no significant differences between the control and PI groups in terms of age (*U* = 3195, *P* = .2918, Fig. 1H), prostatitis duration (*U* = 3388, *P* = .6569, Fig. 1I), pre-therapeutic SAS (*U* = 3084, *P* = .1587, Fig. 1J), SDS (*U* = 3243, *P* = .3669, Fig. 1K), and NIH-CPSI score (*U* = 3017, *P* = .0952, Fig. 1L).

### 3.3. Therapeutic effects of psychological intervention on type III prostatitis

**Figure 2.**
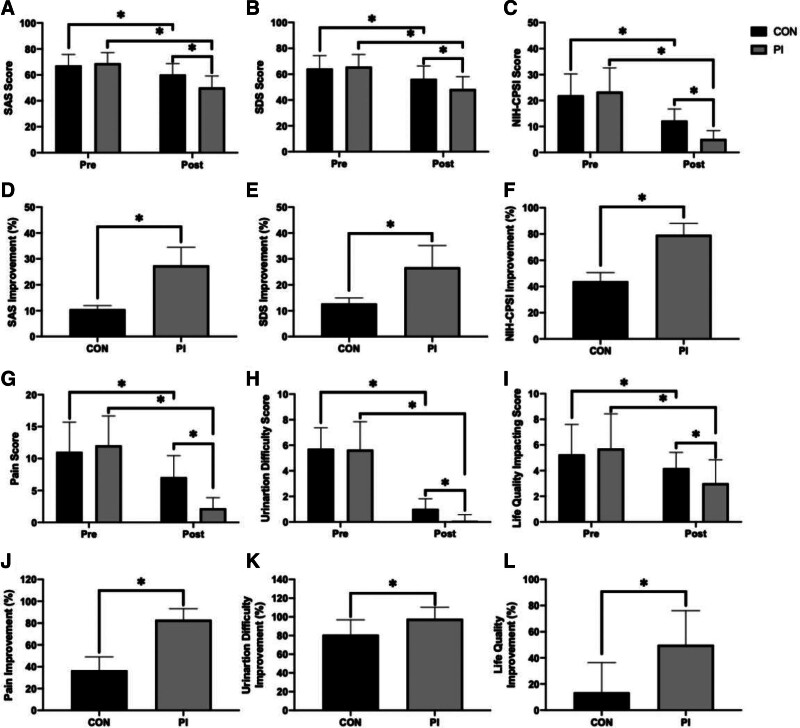
Therapeutic effects on anxiety, depression, severity of prostatitis symptoms and NIH-CPSI scores in the control and psychologically intervened (PI) groups. The pre-therapeutic and post-therapeutic (A) self-rating anxiety scale (SAS), (B) self-rating depression scale (SDS), and (C) National Institutes of Health chronic prostatitis Symptom Index (NIH-CPSI) scores are shown, along with the improvements in (D) SAS, (E) SDS, and (F) NIH-CPSI scores between the control and PI groups. Also presented are the pre-therapeutic and post-therapeutic severity of prostatitis symptoms for (G) pain, (H) urination difficulty, and (I) impact on life quality, as well as the improvements in (J) pain, (K) urination difficulty, and (L) An asterisk () in (A–C) and (G–I) indicates a significant difference (*P* < .05) as determined by repeated-measures 2-way analysis of variance (ANOVA) followed by Sidak post hoc tests; an * in (D–F) and (J–L) indicates a significant difference (*P* < .05) as determined by Mann–Whitney *U* tests. ANOVA = analysis of variance, PI = psychological intervention, SAS = self-rating anxiety scale, SDS = self-rating depression scale, NIH-CPSI = National Institutes of Health-Chronic Prostatitis Symptom Index.

After treatment, both the CON and PI groups showed significantly lower levels on the SAS, SDS, and NIH-CPSI scores. Specifically, for SAS (*F*(1, 166) = 585.0, *P* < .0001; CON: 60.29 ± 8.429, PI: 50.24 ± 8.940), SDS (*F*(1, 166) = 292.8, *P* < .0001; CON: 56.37 ± 10.02, PI: 48.43 ± 9.689), and NIH-CPSI score (*F*(1, 166) = 105.5, *P* < .0001; CON: 12.24 ± 4.511, PI: 5.214 ± 3.227), all showing significant reductions with both *P* < .0001 by 2-way ANOVA-Sidak post hoc analysis (Fig. 2A–C). However, the post-therapeutic levels in the PI group were significantly lower than in the control group for all measures (all *P* < .0001 by 2-way ANOVA-Sidak post hoc, Fig. 2A–C).

Additionally, patients in the PI group showed greater improvement percentages than the control group in SAS levels (CON: 10.58 ± 1.399% vs PI: 27.48 ± 6.993%, *U* = 32, *P* < .0001, Fig. 2D), SDS levels (CON: 12.76 ± 2.184% vs PI: 26.78 ± 8.404%, *U* = 91.5, *P* < .0001, Fig. 2E), and NIH-CPSI scores (CON: 44.08 ± 6.581% vs PI: 79.39 ± 8.691%, *U* = 0, *P* < .0001, Fig. 2F). In terms of prostatitis symptom improvement, both groups showed significant improvement in pain (*F*(1, 166) = 184.0, *P* < .0001), voiding difficulty (*F*(1, 166) = 7.719, *P* < .0001), and life quality (*F*(1, 166) = 48.91, *P* < .0001), with all *P* < .0001 by 2-way ANOVA-Sidak post hoc analysis (Fig. 2G–I). However, the PI group showed greater improvement than the control group in pain relief (CON: 36.77 ± 12.27% vs PI: 83.02 ± 10.13%, *U* = 33.50, *P* < .0001, Fig. 2J), voiding difficulty (CON: 80.97 ± 15.90% vs PI: 97.98 ± 12.30%, *U* = 1106, *P* < .0001, Fig. 2K), and life quality (CON: 13.65 ± 22.76% vs PI: 49.92 ± 26.23%, *U* = 957.5, *P* < .0001, Fig. 2L).

### 3.4. *Influence of pre-therapeutic clinical features and psychological intervention on therapeutic effec*ts

In analyzing the treatment of prostatitis, we incorporated variables that could act as confounding factors or effect modifiers into multiple linear regression models. Table 1 presents *R*-squared (*R*²) values and β-coefficients (with 95% confidence intervals [CI]) for models using post-therapeutic National Institutes of Health-Chronic Prostatitis Symptom Index score (post-NIH-CPSI), the difference in NIH-CPSI score before/after therapy (∆NIH-CPSI, [post-NIH-CPSI]–[pre-NIH-CPSI]), and the improvement percentage of NIH-CPSI score (∆NIH-CPSI (%), −[∆NIH-CPSI]/[pre-NIH-CPSI] × 100%) as dependent variables. The independent parameters included age, duration of prostatitis, pre-therapeutic SAS (pre-SAS), SDS (pre-SDS), NIH-CPSI (pre-NIH-CPSI) scores, and PI. Across all 3 models, significant predictors affecting the dependent variables were the duration of prostatitis, pre-SAS, pre-NIH-CPSI, and PI (all β-coefficients *P* < .05).

**Table 1:** Parameter estimated by multiple linear regression analysis for post-therapeutic (post-NIH-CPS), difference (ANIH-CPSI) and the improvement NIH-CPSI (ANIH-CPSI (%)) scores as dependent variables and age, prostatitis course, pre-therapeutic SAS, SDS and NIH-CPSI scores, and psychological intervention as independent variables.

Dependent variables parameter	Post-NIH-CPSI, *R*² = 0.9394, adjusted *R*² = 0.9372			△NIH-CPSI, *R*² = 0.9640, adjusted *R*² = 0.9627			△NIH-CPSI (%), *R*² = 0.8988, adjusted *R*² = 0.8951		
	β coefficient*	*P*	95% CI	β coefficient*	*P*	95% CI	β coefficient*	*P*	95% CI
Intercept	−2.308 ± 1.465	.1171	−5.201/0.5850	−2.308 ± 1.465	.1171	−5.201/0.5850	72.37 ± 6.943	<.0001	58.65/86.08
A. Age	0.005269 ± 0.01446	.7159	−0.02328/0.03382	0.005269 ± 0.01446	.7159	−0.02328/0.03382	0.1091 ± 0.06851	.1134	−0.02623/0.2444
B. Prostatitis course	0.2062 ± 0.05622	.0003	0.09522/0.3173	0.2062 ± 0.05622	.0003	0.09522/0.3173	−1.183 ± 0.2664	<.0001	−1.710/−0.6572
C. Pre-SAS	0.09869 ± 0.03131	.0019	0.03686/0.1605	0.09869 ± 0.03131	.0019	0.03686/0.1605	−0.6226 ± 0.1484	<.0001	2.777912621
D. Pre-SDS	0.1635 ± 0.02923	.5767	−0.04137/0.07407	0.1635 ± 0.02923	.5767	−0.04137/0.07407	0.1079 ± 0.1385	.4372	−0.1657/0.3815
E. Pre-NIH-CPSI	0.2230 ± 0.02681	<.0001	0.1700/0.2759	−0.7770 ± 0.02681	<.0001	−0.8300/−0.7241	0.6540 ± 0.1271	<.0001	0.4031/0.9049
F. Psychological intervention	−7.624 ± 0.2061	<.0001	−8.031/−7.217	−7.624 ± 0.2061	<.0001	−8.031/−7.217	35.58 ± 0.9767	<.0001	33.65/37.50

CI = confidence intervals, NIH-CPSI = National Institutes of Health-Chronic Prostatitis Symptom Index Scale, post-NIH-CPSI = post-therapeutic National Institutes of Health-Chronic Prostatitis Symptom Index, SAS = self-rating anxiety scale, SDS = self-rating depression scale.

* β Coefficients indicate how much a dependent variable change per each unit variation of the independent variable, taking into account the effect of the other independent variables in the model. Data are shown for 168 prostatitis patients.

In the model predicting post-NIH-CPSI (Table 1), the β-coefficients for prostatitis duration, pre-SAS, and pre-NIH-CPSI were positive, while the β-coefficient for PI was negative. This indicated that patients with longer prostatitis durations, higher anxiety levels, and greater severity of prostatitis before therapy tended to have higher NIH-CPSI scores post-therapy. However, PI was effective in reducing NIH-CPSI scores.

For the ∆NIH-CPSI model (Table 1), the β-coefficients for prostatitis duration and pre-SAS were positive, while those for pre-NIH-CPSI and PI were negative. In the ∆NIH-CPSI (%) model (Table 1), the β-coefficients for prostatitis duration and pre-SAS were negative, and those for pre-NIH-CPSI and PI were positive. These results suggest that patients with longer prostatitis durations and higher anxiety levels experienced less symptom improvement post-therapy, whereas those with higher initial severity of prostatitis and who received PI demonstrated greater symptom improvement. Notably, the β-coefficients for PI in the ∆NIH-CPSI and ∆NIH-CPSI (%) models were substantial enough.

### 3.5. Synergistic effect of psychological intervention with pre-therapeutic clinical features

To investigate how the interaction between 2 variables is influenced by a third variable, we utilized 2-way interaction multiple linear regression. This approach was employed to estimate the synergistic effect of PI with pre-therapeutic clinical features, with a particular focus on the duration of prostatitis. The choice of prostatitis duration as a key clinical feature was based on its significant impact on the dependent variable in the full multiple linear regression model (Table 1) and its notable correlation with the NIH-CHSI score (Fig. 1G).

In the full model of post-NIH-CPSI, when the 2-way interaction of prostatitis duration and PI was included, the β-coefficients for prostatitis duration, pre-SAS, and pre-NIH-CPSI remained positive, while those for PI and the 2-way interaction were negative (Table 2). The inclusion of this 2-way interaction resulted in a better model fit (adjusted *R*² = 0.9521, *F*(1, 160) = 51.0915, *P* < .0001). This indicates that patients with longer prostatitis durations benefited more from PI in terms of symptom improvement.

**Table 2:** Parameter estimated by multiple linear regression analysis combining with simplification and 2-way interactions for post-therapeutic NIH-CPSI score as dependent variable and removement of age and pre-therapeutic SDS score for simplification and 2-way interaction between prostatitis courses and psychological intervention in independent variables.

Dependent variables parameter	Two-way interaction MLR, *R*² = 0.9541, adjusted *R*² = 0.9521			Simplified MLR, *R*² = 0.9393, adjusted *R*² = 0.9378			Two-way interaction simplified MLR, *R*² = 0.9540, adjusted *R*² = 0.9526		
	β coefficient*	*P*	95% CI	β coefficient*	*P*	95% CI	β coefficient*	*P*	95% CI
Intercept	−2.480 ± 1.280	.0544	−5.007/0.04716	−1.903 ± 1.223	.1216	−4.319/0.5118	−2.558 ± 1.071	.0181	58.65/86.08
A. Age	−0.0002630 ± 0.01265	.9834	−0.02524/0.02472	–	–	–	–	–	–
B. Prostatitis course	0.3658 ± 0.05395	<.0001	0.2593/0.4723	0.2219 ± 0.04460	<.0001	0.1339/0.3100	0.3626 ± 0.04352	<.0001	−5.201/0.5850
C. Pre-SAS	0.09785 ± 0.02734	.0005	0.04385/0.1518	0.1066 ± 0.02342	<.0001	0.06035/0.1528	0.09283 ± 0.02052	<.00010	0.09522/0.3173
D. Pre-SDS	−0.007593 ± 0.02575	.7684	−0.05844/0.04325	–	–	–	–	–	–
E. Pre-NIH-CPSI	0.2457 ± 0.02363	<.0001	0.1990/0.2924	0.2279 ± 0.02525	<.0001	0.1780/0.2778	0.2434 ± 0.02214	<.0001	0.03686/0.1605
F. Psychological intervention	−5.099 ± 0.3965	<.0001	−5.882/−4.316	−7.621 ± 0.2043	<.0001	−8.024/−7.218	−5.111 ± 0.3907	<.0001	−8.890598291
B×F	−0.2800 ± 0.03917	<.0001	−0.3574/−0.2026				−0.2786 ± 0.03859	<.0001	1.146250518

CI = confidence intervals, MLR = multiple linear regression, NIH-CPSI = National Institutes of Health-Chronic Prostatitis Symptom Index, post-NIH-CPSI = post-therapeutic National Institutes of Health-Chronic Prostatitis Symptom Index, SDS = self-rating depression scale.

* β Coefficients indicate how much dependent variable changes per each unit variation of the independent variable, taking into account the effect of the other independent variables in the model. Data are shown for 168 prostatitis patients.

On the other hand, the removal of insignificant parameters, such as age and pre-SDS, from the full model resulted in a better-fitted simplified multiple linear regression model (adjusted *R*² = 0.9378, *F*(2, 161) = 0.190895, *P* < .0001, Table 2). Adding the 2-way interaction of prostatitis duration and PI to this simplified model further improved the fit (adjusted *R*² = 0.9526, *F*(1, 162) = 52.1187, *P* < .0001, Table 2). This highlights that the key parameters influencing the model’s predictions are prostatitis duration, pre-SAS, pre-NIH-CPSI, and PI. Especially, patients with longer prostatitis courses demonstrated a marked requirement for PI for further improvement of their symptoms.

Moreover, simplifying the model by removing nonsignificant parameters such as age and pre-SDS resulted in a better-fitting multiple linear regression model (adjusted *R*^2^ = 0.9378, *F*(2,161) = 0.190895, *P* < .0001, Table 2). Reintroducing the 2-way interaction of prostatitis course and PI into this simplified model enhanced the model fit (adjusted *R*^2^ = 0.9526, *F*(1,162) = 52.1187, *P* < .0001, Table 2). This underscores the importance of prostatitis, pre-SAS, pre-NIH-CPSI, and PI as key factors influencing the model predictions. It is important to note that as the prostatitis courses increase, it becomes clearer that PIs can benefit patients with prostatitis and result in improved chronic prostatitis symptoms.

The dependent variable, ∆the NIH-CPSI score, was analyzed using a least squares regression model complemented by cross-tabulation analysis. The goodness of fit for this model was notable with a degree of freedom (DF) of 157, an *R*² = 0.9827, and an adjusted *R*² = 0.9816. The regression analysis highlighted several significant findings. The progression of prostatitis (measured in months) was 5.694, with DF = 1, MS = 5.694, *F*(1, 157) = 6.641, and *P* = .0109 < 0.05. In addition, the pre-SAS score SS was 0.4930 with DF = 1, MS = 0.4930, *F*(1, 157) = 0.5750, and *P* = .4494. The pre-NIH-CPSI score SS was 39.54 with DF = 1, MS = 39.54, *F*(1, 157) = 46.11 and *P* < .0001. Finally, the PI SS was 2398, with DF = 1, MS = 2398, *F*(1, 163) = 1392 and *P* < .0001. The raw data were and the following parameter estimates were obtained for the progression of prostatitis (months): Estimate = 0.1648, Standard Error = 0.06396, 95% CI (asymptotic) ranging from 0.03849 to 0.2911. The *P*-value obtained was significant at a level of <.0001 and the absolute *t*-value was 2.577. In addition, the pre-SAS score estimate was obtained as −0.02544 with a standard error of 0.03355: the asymptotic 95% confidence interval ranges from −0.09170 to 0.04083 with |*t*| = 0.7583 and *P* = .4494. The pre-NIH-CPSI score estimate is −0.2445 with a standard error of 0.03600 and an asymptotic 95% confidence interval of −0.3156 to −0.1734, |*t*| = 6.790, *P* < .0001. Duration of prostatitis (months): the pre-SAS score was SS = 8.523, DF = 1, MS = 8.523, *F*(1157) = 9.940, and *P* = .0019 < 0.05. The estimate for PI is −1.777 with a standard error = 1.764. The 95% CI (asymptotic) ranges from −5.261 to 1.706, with a |*t*| value of 1.008 and *P* = .3151.

Furthermore, the multiple covariance analysis indicated that prostatitis duration had a variance inflation factor (VIF) of 25.89 with an *R*² = 0.9614, and the pre-SAS score had a VIF = 269.5 with an *R*² = 0.9963. The pre-NIH-CPSI evaluation score had a VIF = 45.47 and an *R*² = 0.9780, while PI had a VIF = 152.4 and an *R*² = 0.9934. The model analysis revealed a correlation between the previous NIH-CPSI score and the duration of chronic prostatitis. Cross-analysis, while excluding confounding factors, indicated that longer disease duration correlates positively with the pre-SAS score and negatively with the effectiveness of PIs. The pre-NIH-CPSI scores increased with a longer duration of prostatitis, suggesting that early PI is advisable for patients with CP/CPPS.

## 4. Discussion and conclusion

This study revealed that older patients with prostatitis generally experienced longer disease courses, higher levels of anxiety and depression, and greater disease severity. Notably, patients with prolonged prostatitis also exhibited increased levels of anxiety, depression, and disease severity. While standard prostatitis pharmacotherapy proved effective, PI significantly enhanced its effects on reducing anxiety, depression, pain, voiding difficulty, life quality, and overall disease severity in CP/CPPS patients. Key pre-therapeutic clinical features influencing therapeutic efficacy in CP/CPPS included the duration of prostatitis, anxiety levels, and disease severity, but not age or depression levels. A notable finding was the synergistic effect between PIs and the duration of prostatitis, indicating that patients with longer disease courses particularly benefitted from PIs.

The findings of this study are in line with previous studies which state that patients with longer prostatitis courses also had higher anxiety and depression levels and disease severity.^[24,32,33]^ Although standard prostatitis pharmacotherapy was effective,^[34,35]^ the PI had been demonstrated to enhance the improve efficacy on improving the anxiety, depression, pain relief, voiding difficulty, life quality, and disease severity of patients with CP/CPPS.^[36]^ The efficacy of prostatitis treatment is primarily affected by the course of prostatitis, anxiety level, and severity of prostatitis in CP/CPPS patients. Age and degree of depressive symptoms have a minimal impact. Additionally, PI has a synergistic effect when combined with prostatitis treatment. Timely PI is particularly important for patients with a longer course of prostatitis.

Previous research has reported a strong association between anxiety disorders and CP/CPPS in a population-based study.^[9]^ The etiology of CP/CPPS, distinct from type I and II prostatitis caused by identifiable prostatic infections, involves an imbalance between pro-inflammatory and anti-inflammatory cytokines, implicated in pelvic pain.^[37]^ This condition might be influenced hormonally through androgen receptor defects.^[38]^ The prostate has been considered a source of chronic pelvic pain symptoms.^[39]^ Likely due to correlations with neurogenic inflammation and central sensitization from nerve growth factors.^[40,41]^ Anxiety is also known to be associated with other autoimmune diseases’ progression,^[42]^ androgen receptor inactivation,^[37]^and nerve growth factor release.^[43–46]^ PI is generally acknowledged as an effective therapy for anxiety disorders.^[47,48]^ Therefore, combining PI with standard prostatitis pharmacotherapy is believed to be clinically effective for CP/CPPS. This approach aligns with the efficacy found in other studies.^[10,49,50]^

Although PI has been mentioned as having high clinical efficacy in conjunction with standard prostatitis pharmacotherapy in several previous studies, they rarely pointed out which CP/CPPS patients required PI the most. In our study, standard prostatitis pharmacotherapy, consisting of tamsulosin once per day for 30 days and levofloxacin twice per day for 10 days, was effective in improving the symptoms of CP/CPPS. However, the standard prostatitis pharmacotherapy showed weaker efficacy in CP/CPPS patients with longer disease courses. It was generally believed that CP/CPPS patients with longer disease courses would have higher anxiety levels, a situation also observed in our results. It was generally believed that the CP/CPPS patients with longer disease courses would have higher anxiety levels^[28]^ and this situation was also observed in our results. Unfortunately, we observed that the standard prostatitis pharmacotherapy could not adequately address the rising anxiety levels. Although tamsulosin was useful in improving nocturia caused by anxiety,^[51]^ neither tamsulosin nor levofloxacin was effective in dealing with anxiety. Furthermore, levofloxacin has been reported to induce anxiety and insomnia in young adults.^[49]^

Since anxiety may be a key factor in the pathogenesis of CP/CPPS,^[9]^ the CP/CPPS patients with longer disease courses who also have higher anxiety levels required more efficient therapy than the standard prostatitis pharmacotherapy to deal with their CP/CPPS and high anxiety levels. In our results, PI combined with standard prostatitis pharmacotherapy was the high-efficiency therapy for CP/CPPS with a longer disease course. PI combined with pharmacotherapy, tamsulosin, and levofloxacin, was the high-efficiency therapy for CP/CPPS with a longer disease course in our results. However, several other methods were tried to deal with the decrease of anxiety levels in CP/CPPS patients, including oral corticosteroid therapy.^[51]^ In that study, oral corticosteroid therapy did not improve the anxiety or deterioration of the anxiety levels or NIH-CPSI scores. Comparing our results with that previous research, PI was the better way to promote recovery from anxiety disorder in CP/CPPS. PI is considered an efficient method to recover from anxiety disorder or serious psychological issues, such as post-traumatic stress disorder.^[36]^ Additionally, it has been used to relieve chronic pain and improve the quality of life for other illnesses.^[52,53]^ Therefore, the significant impact of life quality, chronic pain, and anxiety disorder on patients with prolonged CP/CPPS courses requires a combination of PI and standard pharmacotherapy for further and more significant therapeutic effects.

PI, combined with pharmacotherapy, tamsulosin, and levofloxacin, was the high-efficiency therapy for CP/CPPS with a longer disease course in our results. However, several other methods were tried to deal with the decrease of anxiety levels in CP/CPPS patients, including oral corticosteroid therapy.^[51]^ In that study, oral corticosteroid therapy did not improve the anxiety or deterioration of the anxiety levels or NIH-CPSI scores. Comparing our results with that previous research, PI was the better way to promote recovery from anxiety disorder in CP/CPPS. PI is considered an efficient method to recover from anxiety disorder or serious psychological issues, such as post-traumatic stress disorder.^[36]^ Besides, it was also used to relieve chronic pain and improve life quality from other illnesses.^[52,53]^ Therefore, the course of prostatitis has a significant impact on the quality of life, chronic pain, and anxiety disorders of CP/CPPS patients, but the use of PI in combination with standard drug treatment may have better therapeutic effects than treatment with the standard prostatitis drug alone.

Although PIs have therapeutic benefits for CP/CPPS, there are cost issues and certain limitations to consider. For instance, while the total treatment duration for CP/CPPS is not necessarily longer, patients might need to invest more time and money in psychotherapy compared to standard prostatitis medication alone. Patients with CP/CPPS are more likely to suffer from depression and anxiety, and psychological factors significantly impact the severity of prostatitis symptoms and quality of life. Currently, PI can effectively address these issues. Furthermore, it positively influences chronic prostatitis^[21]^ and does not have significant negative effects on the clinical management of chronic pelvic pain syndrome (CP/CPPS). Our study findings indicate that the longer the prostatitis course, the higher the patient’s SAS score, SDS score, and National Institutes of Health chronic prostatitis Symptom Index (NIH-CPSI) score. Additionally, implementing PI in the early stages of the disease can shorten its course and alleviate both physical and psychological symptoms. However, as prostatitis progresses, the efficacy of PI gradually diminishes. Therefore, we recommend that patients with chronic prostatitis or chronic pelvic pain syndrome who have a prolonged course of prostatitis and high symptom scores should receive PI as soon as possible, alongside standard prostatitis medication. This comprehensive treatment approach enhances the effectiveness of the therapy and accelerates the patient’s recovery, facilitating a quicker return to normal daily activities.

In conclusion, this study provides the first evidence supporting the integration of PIs with pharmacotherapy for alleviating multidimensional symptoms (pain, voiding dysfunction, and psychological distress) in CP/CPPS. The identification of anxiety levels and social support networks as key moderators of treatment response advances personalized therapeutic strategies for this complex syndrome. However, several limitations should be acknowledged. Firstly, recruiting patients from only 1 hospital-controlled healthcare system confounding factors, enhancing internal effectiveness. However, it reduced racial and socio - economic diversity, limiting cross-cultural universality and international collaboration. Secondly, the moderate sample size (\(n = 168\)) precluded robust subgroup analysis for differences in age groups or comorbidity features. Thirdly, while SAS/SDS is reliable, the lack of cross-cultural validation of psychological assessment tools in our cohort may conceal culture-mediated differences in symptom reports. Finally, our machine learning model focused on interpretable clinical variables like baseline anxiety, omitting neuroimaging or biomarker data that could show brain-gut-peripheral interactions.

## Author contributions

**Conceptualization:** Xiu Lin, Shih-Pin Lee.

**Data curation:** Xiu Lin, Xian-Bin Pan, Shih-Pin Lee.

**Formal analysis:** Xiu Lin.

**Funding acquisition:** Xiu Lin.

**Investigation:** Xiu Lin, Xian-Bin Pan, Bao-Zhu Guo.

**Methodology:** Xiu Lin, Shih-Pin Lee.

**Project administration:** Xiu Lin, Shih-Pin Lee.

**Resources:** Xiu Lin.

**Software:** Xiu Lin.

**Supervision:** Shih-Pin Lee.

**Validation:** Shih-Pin Lee.

**Visualization:** Xiu Lin, Shih-Pin Lee.

**Writing** – **original draft:** Xiu Lin.

**Writing** – **review & editing:** Shih-Pin Lee.
